# An efficient autometallography approach to localize lead at ultra-structural levels of cultured cells

**DOI:** 10.1007/s41048-020-00116-9

**Published:** 2020-10-31

**Authors:** Han Song, Gang Zheng, Xue-Feng Shen, Zai-Hua Zhao, Yang Liu, Yang Liu, Ying-Ying Liu, Jun-Jun Kang, Jing-Yuan Chen, Wen-Jing Luo

**Affiliations:** 1 Department of Health Service, PLA General Hospital, Beijing 100853, China; 2 Department of Occupational and Environmental Health and the Ministry-of-Education’s Key Laboratory of Hazard Assessment and Control in Special Operational Environment, School of Public Health, Fourth Military Medical University, Xi’an 710032, China; 3 Department of Neurology, Xijing hospital, Fourth Military Medical University, Xi’an 710032, China; 4 Institute of Neurosciences, Fourth Military Medical University, Xi’an 710032, China

**Keywords:** Lead, Autometallography, Subcellular fraction, Energy-dispersive X-ray spectroscope, Electron microscopy

## Abstract

Understanding the precise intracellular localization of lead (Pb) is a key in deciphering processes in Pb-induced toxicology. However, it is a great challenge to trace Pb *in vitro*, especially in cultured cells. We aimed to find an innovative and efficient approach to investigate distribution of Pb in cells and to validate it through determining the subcellular Pb content.

We identified its ultra-structural distribution with autometallography under electron microscopy in a choroidal epithelial Z310 cell line. Electron microscopy in combination with energy-dispersive X-ray spectroscope (EDS) was employed to provide further evidence of Pb location. In addition, Pb content was determined in the cytosol, membrane/organelle, nucleus and cytoskeleton fractions with atomic absorption spectroscopy.

Pb was found predominantly inside the nuclear membranes and some was distributed in the cytoplasm under low-concentration exposure. Nuclear existence of Pb was verified by EDS under electron microscopy. Once standardized for protein content, Pb percentage in the nucleus fraction reached the highest level (76%).

Our results indicate that Pb is accumulated mainly in the nucleus of choroid plexus. This method is sensitive and precise in providing optimal means to study the ultra-structural localization of Pb for *in vitro* models. In addition, it offers the possibility of localization of other metals in cultured cells. Some procedures may also be adopted to detect target proteins via immunoreactions.

## INTRODUCTION

Neurotoxicity due to lead (Pb) accumulation in specific brain areas causes a wide variety of symptoms including decreased intelligence quotient, cognitive deficits, poor attention span and increased aggression (Boucher *et al*. 2012; Calderón *et al*. 2001; Needleman *et al*. 1996; Tong *et al*. 1998). The mechanism of Pb-induced disturbance of neuronal functions included interfering with neurotransmitter release, disrupting the function of GABAergic, dopaminergic, and cholinergic systems, inhibiting N-methyl-D-aspartic acid receptor (NMDAR) and NMDAR-mediated signaling pathways that are necessary for learning, memory and synaptic plasticity (Akinyemi *et al*. 2019; Basha *et al*. 2012; Wang *et al*. 2016; Wirbisky *et al*. 2014).

The pathways by which Pb enters into the brain parenchyma are attributed to the breakdown of brain barriers including the blood-brain barrier (BBB) and blood-cerebrospinal fluid (CSF) barrier (BCB) (Shi and Zheng 2007; Song *et al*. 2014; Wang *et al*. 2007). In studying Pb-induced toxicity in the BCB, *in vitro* experiments have played a crucial role in delineating its breakdown, inhibition of production and secretion of transthyretin and accumulation of beta-amyloid (Behl *et al*. 2010; Shi and Zheng 2007; Zheng *et al*. 1999). Additionally, manipulations of nucleic acid levels or protein kinase activity in *in vitro* models, especially in cell cultures, have helped to unravel key regulatory molecules associated with Pb’s effects. In our previous study on Pb-induced breakdown of the BBB in rat brain microvascular endothelial RBE4 cells, reduction of brain barrier tight junctional proteins was regulated by activation of tyrosine kinase Src via chaperon glucose-regulated protein of 78 kDa (GRP78) (Song *et al*. 2014). However, the intracellular Pb localization has been ignored in exploring the molecular mechanisms of Pb toxicity.

Approaches of Pb localization in cells may be classified into three modalities: intuitive observation in combination with energy-dispersive X-ray spectroscope (EDS), isotopic tracer labeling, and autometallography (AMG). In the 1970s, high-dose Pb was used in numerous studies. This metal was visualized directly as nuclear inclusion bodies in renal tubular cells of rats (Moore and Goyer 1974). Cytoplasmic or intranuclear inclusions observed in macrophages and astrocytes of Pb-implanted rat brains were further studied with EDS (Shirabe and Hirano 1977). To localize Pb in the developing rat cerebellum, autoradiographs were prepared by both light microscopy and electron microscopy from cerebellar tissues after injection of radioactive Pb (^210^Pb) (Thomas *et al*. 1973). Another radioactive Pb isotope, ^203^Pb, was used to test the binding capacity of nuclear and inclusion body fractions induced by Pb pretreatment (Oskarsson and Fowler 1985). AMG, an extremely precise and sensitive metal tracing cytochemical technique, has been used to detect gold, silver, mercury, bismuth or zinc (Zn) with sulfur and/or selenium (Danscher and Stoltenberg 2006). Electrons released from hydroquinone molecules adhere to the metal sulfides or metal selenides. The silver ions are attracted to catch the electrons and aggregate around the metals as silver atoms, which can be visualized as black silver deposits (BSDs) (Danscher and Møller-Madsen 1985). Under light microscopy, BSDs were found in the frontal epithelium of the gill filament in the Pb-exposed mussels (Domouhtsidou and Dimitriadis 2000). BSDs also existed in the dense bodies of the epithelial cells in *Mytilus galloprovincialis* under electron microscopy after 30- and 60-day Pb exposure (Dimitriadis *et al*. 2003). To our knowledge, methods to localize Pb in cultured cells have yet to be developed.

The choroid plexus, a highly vascularized tissue, constitutes the BCB. One side of the BCB confronts blood circulation, and the other side faces CSF isolated from blood stream in cerebral compartments. As a barrier between the blood and CSF, choroid plexus not only plays a vital role in regulating the homeostasis of the central nervous system by rigorously restricting access of substances from the blood to the CSF, but also facilitates neuronal development by producing and secreting growth factors, peptides, neurotrophins, hormones, and protein stabilizers, such as transthyretin (Chodobski and Szmydynger-Chodobska 2001; Levine 1987; Zheng 2001). Choroid plexus was the target for many heavy metals including Pb in previous studies. Pb level increased with age in human choroid plexus (Friedheim *et al*. 1983). Compared with brain cortex, 100 times increment of Pb was reported in human choroid plexus (Manton *et al*. 1984). Moreover, radiolabeled lead nitrate (^210^Pb) was concentrated about >70 times in choroid plexus than brain of adult pigs (O’Tuama *et al*. 1976). Furthermore, accumulation of Pb was both dose-dependent and time-related in an acute Pb exposure study (Zheng *et al*. 1991). Thus, choroid plexus, not other tissues, was chosen as the research target because of its highest power of Pb accumulation.

A choroidal epithelial Z310 cell line has been established from murine choroid plexus (Zheng and Zhao 2002). The purpose of the present study was to develop a novel approach to investigate the localization of Pb in Z310 cells. The results from this study provide additional evidence for nuclear Pb in mediating BCB toxicity.

## RESULTS

### Pb localization in ultra-structural levels

**Figure 1:**
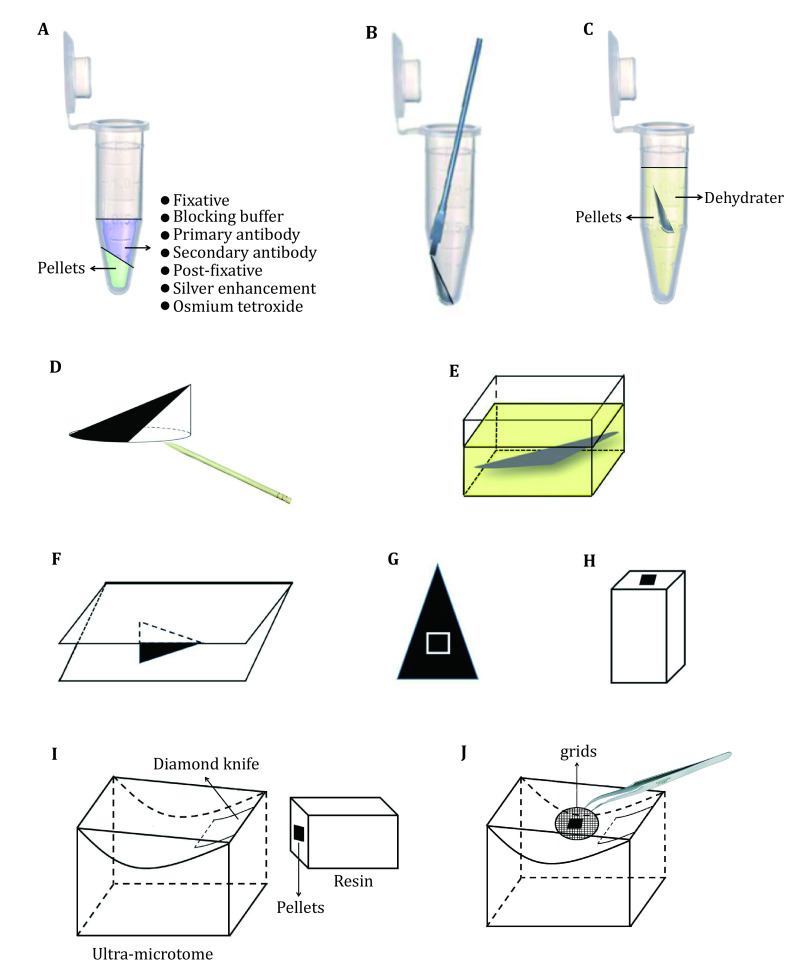
Additional illustration of operational skills. **A** Incubation with pre-fixative, blocking buffer, primary antibody, secondary antibody, post-fixative, reagent of silver enhancement and osmium tetroxide. **B** Detachment from above of the pellets. **C** Dehydration with graded ethanol and acetone. **D** Transfer via the pellets’ bottom with toothpicks. **E** Immersion in soak solution. **F** Placement between two boards and aggregation of embedding medium. **G**, **H** Cut of small pieces from the pellets indicated by the white square in the black triangle (**G**) and stickup on the resins (**H**). **I** Ultrathin sections by the diamond knife of ultramicrotome. **J** Transfer the sections with the grids squeezed by tweezers

**Figure 2:**
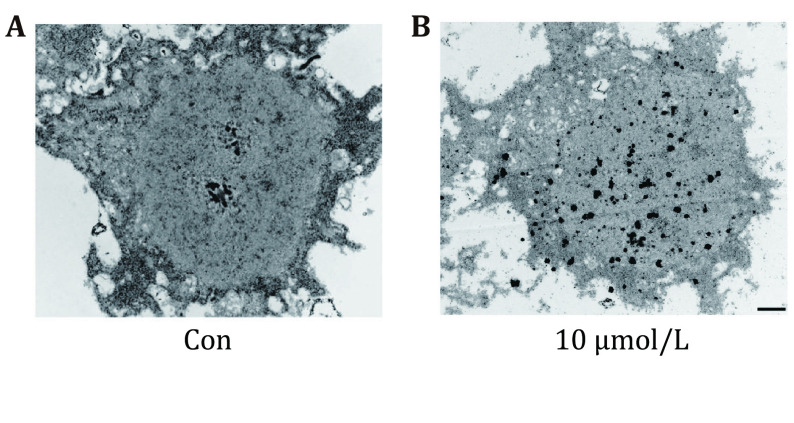
Pb localization detected by electron microscopic AMG staining in Z310 cells after treatment of 0 (Con) (**A**) or 10 μmol/L of Pb (**B**) for 24 h. Scale bars, 1 μm

**Figure 3:**
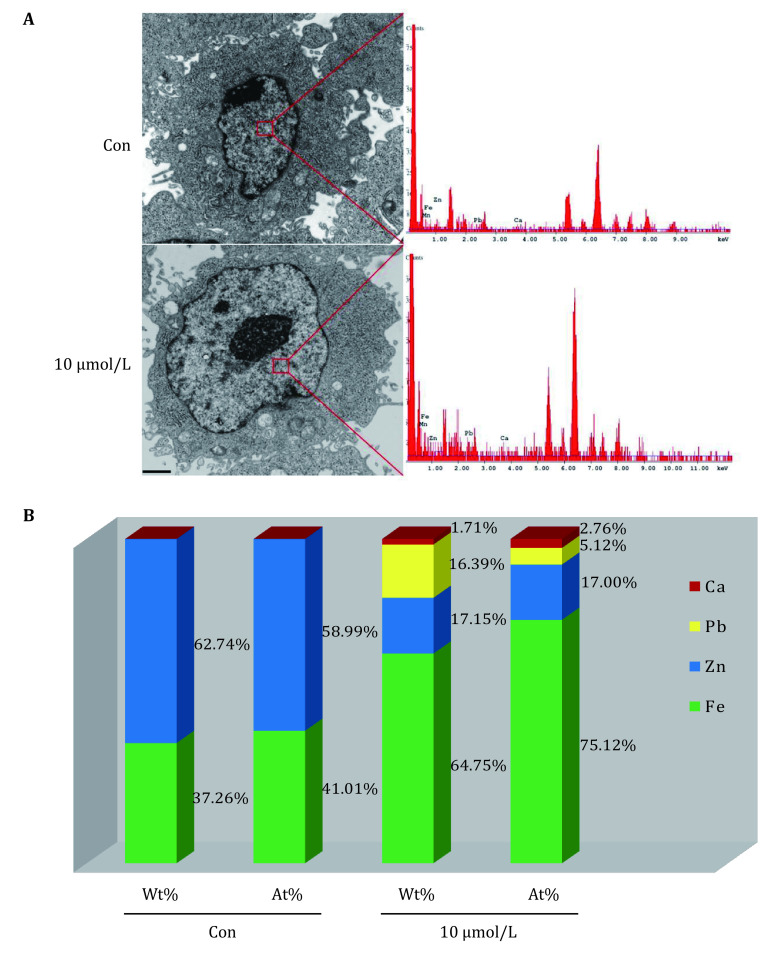
EDS analysis of the nucleus in Z310 cells treated with low-dose Pb. **A** Cell morphology under TEM after treatment of 0 (Con) or 10 μmol/L of Pb for 24 h (left). EDS analysis of nuclear areas marked with the red frames (right). Scale bars, 1 μm. **B** Calculation of Wt% and At% of Pb, Mn, Fe, Zn and Ca in controlled and 10 μmol/L Pb groups respectively

The choroidal epithelial Z310 cell line was used as choroids plexus *in vitro* model. 10 μmol/L was chosen as a working concentration for Pb exposure (Shi and Zheng 2007). To visualize the cellular distribution of Pb upon 10 μmol/L treatment in Z310 cells, we employed electron microscopic AMG technique and recorded the key steps (Fig. 1). Interestingly, particles were found predominantly inside the nuclear membranes and some of them were distributed in the cytoplasm (Fig. 2). As expect, nuclear accumulation of Pb were not observed in 10 μmol/L Pb-treated cells under TEM without AMG staining. The “invisible” Pb in the nucleus, however, could be detected by EDS (Fig. 3A). Wt% and At% of five metal elements including Pb, manganese (Mn), iron (Fe), Zn and calcium (Ca) were calculated simultaneously. In the control group, the nuclear area outlined with red square frame consisted of Fe (Wt%, 37.26%; At%, 41.01%) and Zn (Wt%, 62.74%; At%, 58.99%). In the 10 μmol/L group, the area consisted of four elements including Fe, Zn, Pb and Ca. Pb content (Wt%, 16.39%; At%, 5.12%) was far lower than Fe (Wt%, 64.75%; At%, 75.12%) and Zn (Wt%, 17.15%; At%, 17%). The weight of Zn decreased from 62.74% to 17.15%, but Fe increased from 37.26% to 64.75% (Fig. 3B).

**Figure 4:**
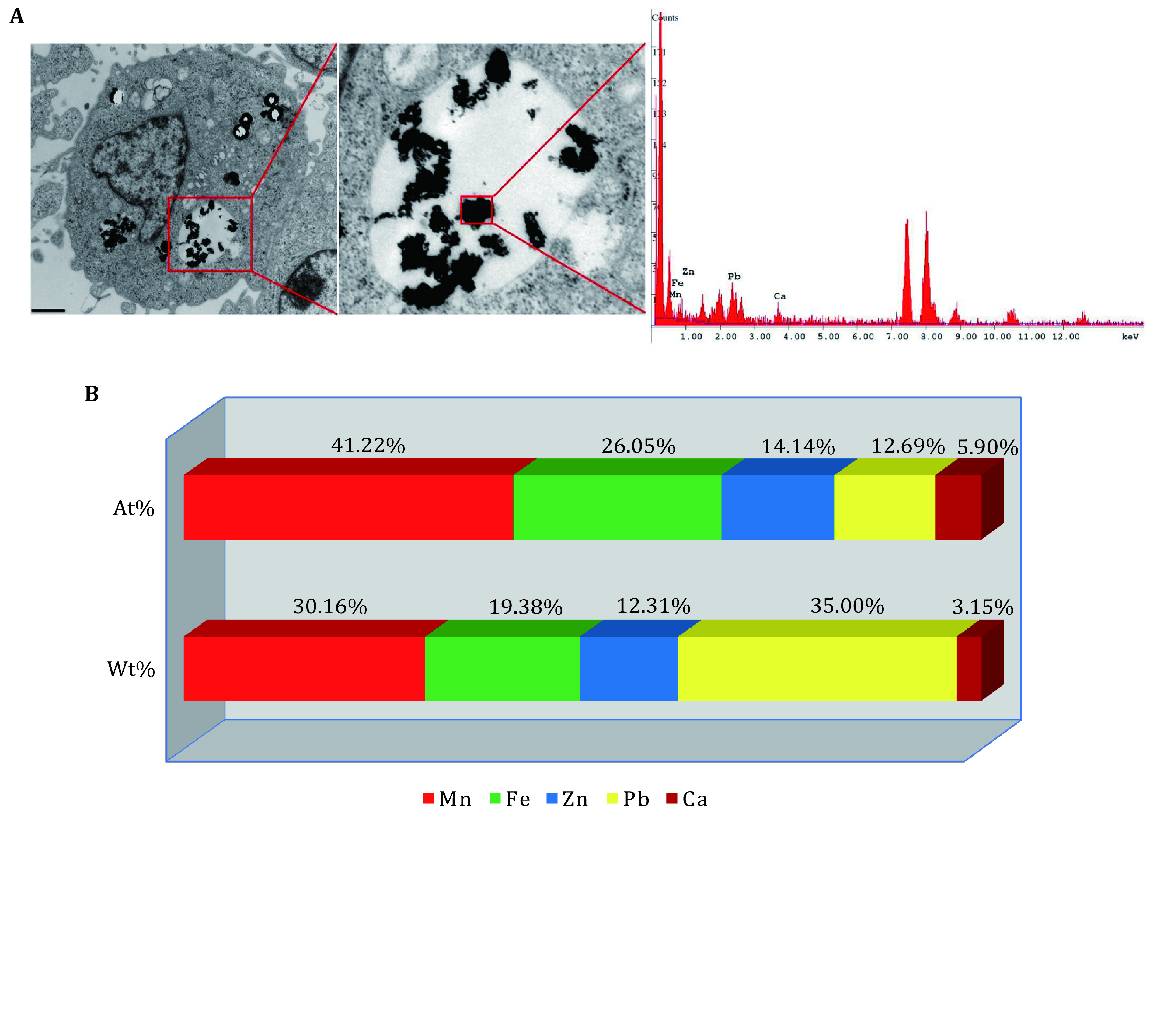
EDS analysis of high-dose Pb-induced particles in the lysosomes. **A** Cellular localization of 200 μmol/L Pb monitored by electron microscopy. EDX analysis (right) of the large particles with high density outlined by red frame (middle) in the lysosome. The lysosome was also framed by the red line in the original cell (left). Scale bars, 1 μm. **B** Calculation of Wt% and At% of Pb, Mn, Fe, Zn and Ca in the indicated area

To verify the EDS analyses of low-level Pb exposure, 200 μmol/L, which reduces cell viability significantly, was chosen as positive control. After 24-h treatment, large particles with high density contained in the lysosome were analyzed by EDS (Fig. 4A). For At%, Mn, Fe, Zn, Pb and Ca accounted for 41.22%, 26.05%, 14.14%, 12.69% and 5.09%, respectively. For Wt%, Mn, Fe, Zn, Pb and Ca accounted for 30.16%, 19.38%, 12.31%, 35% and 3.15%, respectively (Fig. 4B).

### Pb measurement in subcellular fractionation

**Figure 5:**
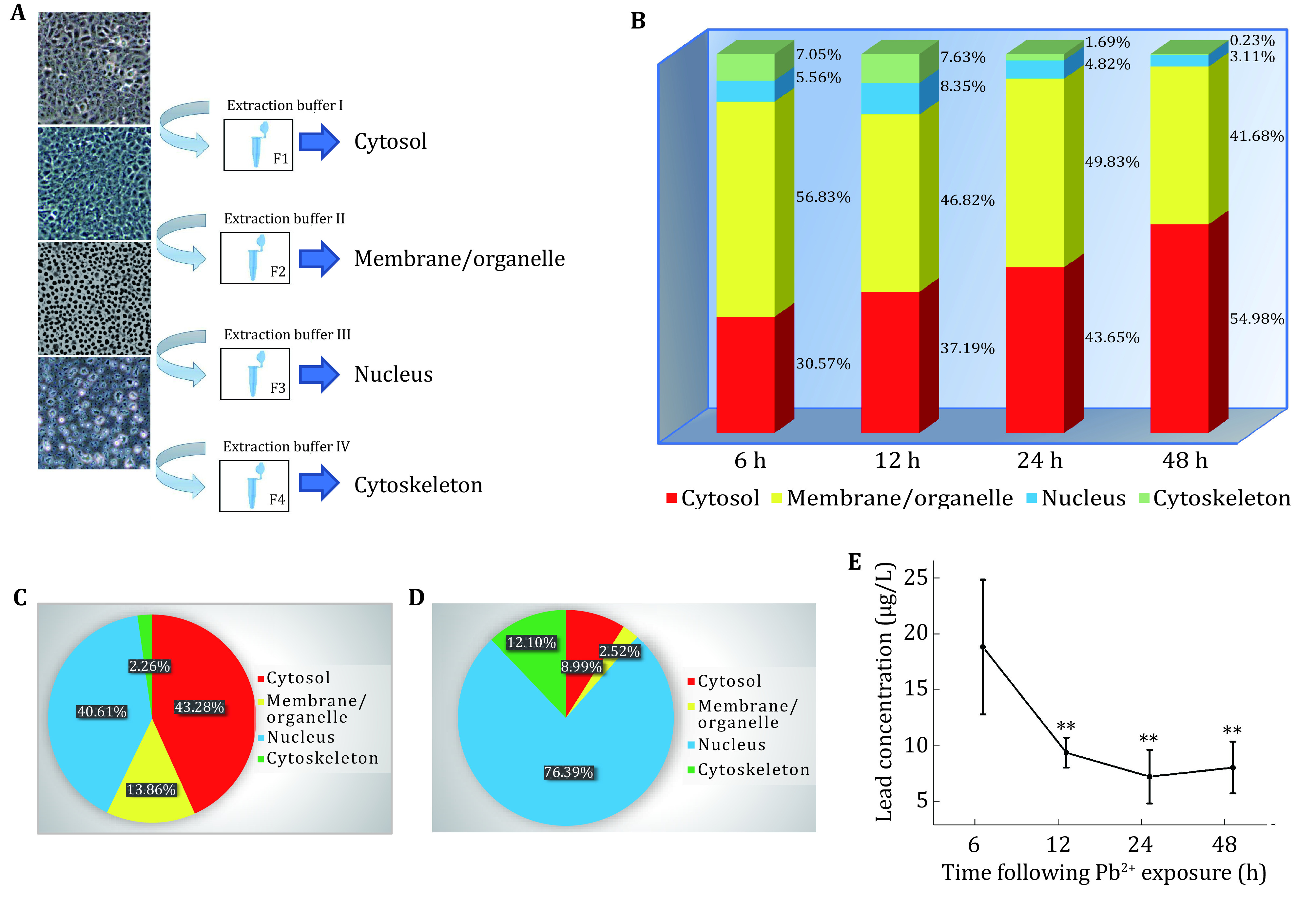
Pb and protein content in subcellular distributions. **A** The process of extraction of cytosol, membrane/organelle, nucleus and cytoskeleton fractions in Z310 cells. The cell morphology was recorded before each step. **B** Hundred-percent barchart of proteins in four fractions 6, 12, 24 or 48 h after Pb exposure. **C** Pie chart of Pb content in such fractions after 24 h exposure. **D** Pie chart of Pb percentage justified by the protein amount after 24 h exposure. **E** Pb concentrations in the nucleus fraction in Z310 cells treated with 10 μmol/L of Pb for 6, 12, 24 and 48 h. Asterisks depict a statistically significant difference (*p* < 0.01) compared with 6 h data ( **E**) with one-way ANOVA. Data shown are representative of three independent experiments

To verify the findings in electron microscopic AMG staining, we next investigated Pb content in subcellular fractions of Z310 cells. Firstly, cellular morphology was captured under the light microscopy before extraction of cytosol, membrane/organelle, nucleus and cytoskeleton fractions (Fig. 5A). Protein content was measured in these four fractions 6, 12, 24 or 48 h after Pb exposure. Protein percentage of cytosol and membrane/organelle fractions was larger than nucleus and cytoskeleton (Fig. 5B). However, Pb was mostly accumulated in cytosol (43%) and nucleus (41%) fractions at 24 h (Fig. 5C). Once corrected for protein content, Pb percentage in the nucleus fraction reached the highest level (76%) (Fig. 5D). In the nucleus fraction, Pb decreased to a lower level at 12 h after reaching the peak at 6 h and was maintained in a relatively unchanged condition from 12 h to 48 h (Fig. 5E). The effectiveness of this novel approach was further verified by the evidence of nuclear Pb accumulation from quantitative perspectives.

### Localization of molecules in ultra-structural levels

**Figure 6:**
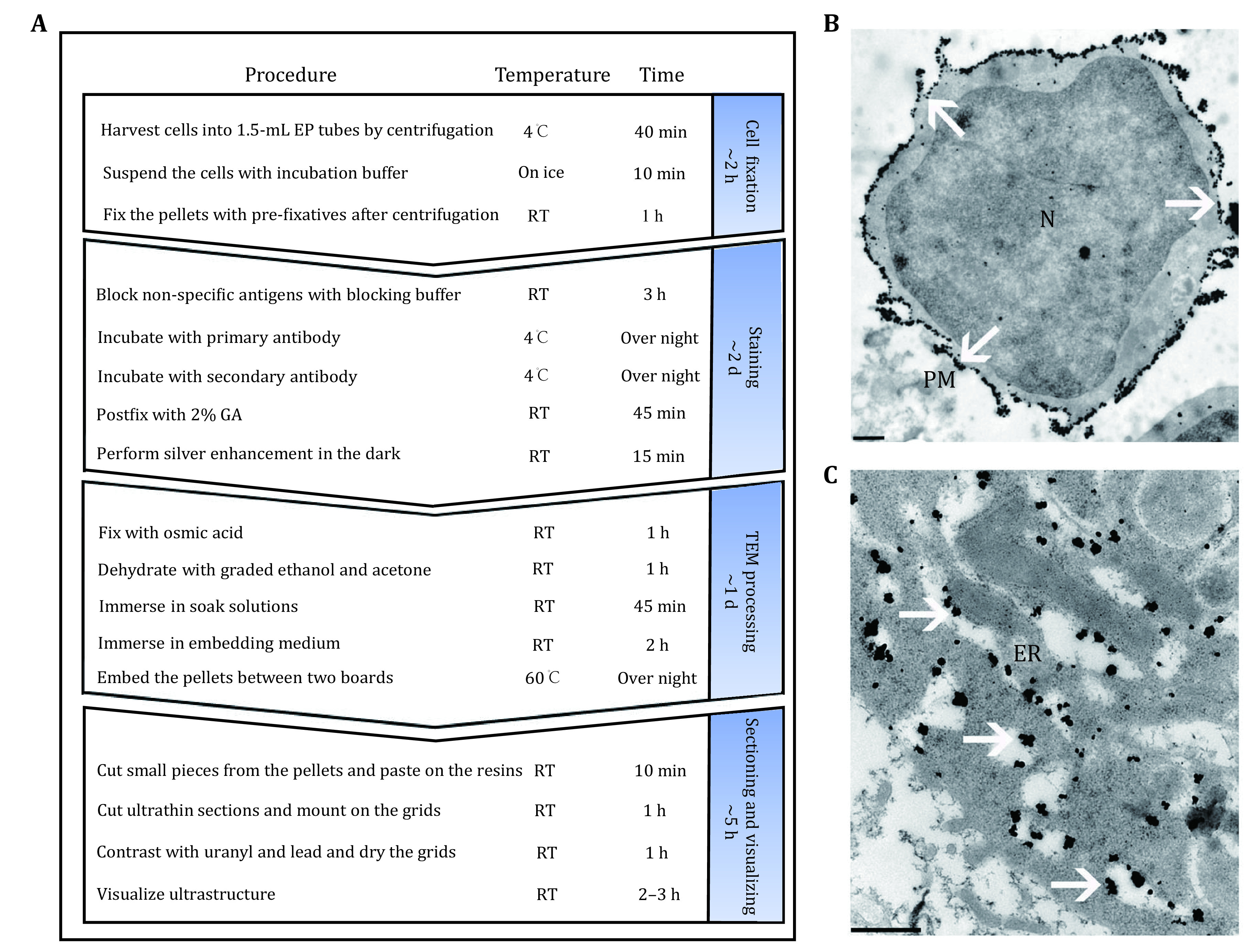
**A** Flow chart with both time and temperature needed in the main procedures of the four parts. RT: room temperature. GA: glutaraldehyde. **B** CD20 localization in human B-lymphocytes. Mouse monoclonal anti-CD20 antibody and goat anti-mouse IgG conjugated to 1.4 nm gold particles were used as the primary and secondary antibody respectively. White arrows point to the localization of CD20. “N” represents nucleus and “PM” represents plasma membrane. **C** GRP78 localization in RBE4 cells. Rabbit polyclonal anti-GRP78 antibody and goat anti-rabbit IgG conjugated to 1.4 nm gold particles were used as the primary and secondary antibody respectively. White arrows point to the localization of GRP78. “ER” represents endoplasmic reticulum. Scale bars, 0.5 μm

Meanwhile, this protocol may also be used as a reference for immunogold-silver staining. Detailed procedures, addressing both temperature and time requirements are shown in the flow chart (Fig. 6A). We determined the localization of CD20 in the B-lymphocytes from human peripheral venous blood (Fig. 6B) and GRP78 in RBE4 cells (Fig. 6C). CD20 was exclusively expressed on the surface of B-lymphocytes, while GRP78 was localized to the lumen of endoplasmic reticulum.

## DISCUSSION

AMG is an effective and precise cytochemical technique to study the accumulation of metals in an organ and its cells. Although AMG was widely used in previous studies to visualize the distribution of metals, AMG granules in cultured cells are difficult to localize under electron microscopy, because junctional molecules fail, in comparison to tissue samples, to provide a three-dimensional structure. In the current study, we investigated the effectiveness of a novel approach on the localization of low-level Pb in cultured cells at ultra-structural level. Based on our previous experiences in carrying out immunogold staining techniques for cell samples (Song *et al*. 2014; Kang *et al*. 2013), we designed this simple and efficient pre-embedding AMG method that does not require special devices or freezing techniques. In the present study, we found that Pb mainly accumulated in the nucleus of the established choroidal epithelial cell line. The newly developed AMG protocol is sensitive and precise in providing optimal means to study the ultra-structural localization of Pb in cultured cells. In addition, some key procedures may also be referred to detect target proteins via immunoreaction in cultured or isolated cells under TEM.

Using this approach, we found nucleus seemed to be an important container for Pb. To further validate the phenomenon, the intracellular Pb distribution was directly determined in relatively well-isolated subcellular fractions. Total nuclear Pb was lower than that in the cytosol, but higher than that in other fractions. After standardization for protein levels, the percentage of Pb content in the nucleus was the highest, indicating that the affinity of Pb to nuclear proteins was significantly higher than the other fractions. Meanwhile, ^54^Mn, a radioactive tracer, was utilized to quantify Mn content in cell extracts after cellular extraction and found to be also present in the nuclei of Z310 cells (Kalia *et al*. 2008). Thus, we posited that nucleus may be the habitat for many heavy metals, not just for Pb. However, a relatively small amount of heavy metals in other fractions should not rule out the importance of some organelle in metal-induced toxicity.

Initially, Pb was found to exist in Pb-protein complexes of intranuclear inclusion bodies in various cells in the 1970s (Goyer *et al*. 1970; Moore and Goyer 1974; Shirabe and Hirano 1977). A major protein, p32/6.3, was identified in the Pb-induced intranuclear inclusion bodies in kidney tubule-lining cells (Shelton and Egle 1982). Furthermore, the increased p32/6.3 under Pb exposure in mouse neuroblastoma 2a (Nb2a) cells was attributed to decreased rate of degradation rather than increased transcription or translation (Klann and Shelton 1989). Our results, though, revealed nuclear Pb accumulation at 10 μmol/L level, no obvious structures like inclusion bodies were observed in this area. Interestingly, the “invisible” Pb could be detected with EDS technique and further confirmed by AMG staining. Apparent discrepancies between our findings of cytoplasmic lysosomes packaging xenobiotics with high electron density under 200 μmol/L Pb exposure and intranuclear inclusion bodies in earlier studies may be due to tissue variability. The constituent ratio of Mn and Pb among such five elements took the majority in the black dots of the lysosomes through analysis of EDS. In a recent study, α-synuclein was found to be regulated by metallothionein, a component of Pb-induced inclusion bodies (Zuo *et al*. 2009). Moreover, α-synuclein served a binding protein for divalent metal ions including Fe and Mn (Binolfi *et al*. 2006). Thus, co-existence of Mn, Pb, even Fe and Zn in the lysosomes in our study may derive from some protein with high-affinity to divalent metal ions.

In summary, the present study proposes a novel approach to localize ultra-structural Pb in cultured cells. What needs to be further investigated is its effectiveness in studying other heavy metal ions in different cells. We hope that this approach may be of reference for colocalization study of proteins and heavy metal ions in future studies of metal toxicology.

## MATERIALS AND METHODS

### Materials

Pb acetate (Pb(AC)_2_) was obtained from Sigma (Saint Louis, MO, USA). Dulbecco’s modified Eagle’s medium (DMEM), RPMI 1640 medium, isometric lymphocyte separation medium, alpha minimum essential medium (α-MEM) with _L_-glutamine, Ham’s F10 (H-F10) with _L_-glutamine, geneticin (G418), penicillin, streptomycin, gentamycin and 0.25% trypsin-EDTA were purchased from Invitrogen (Carlsbad, CA, USA). Basic fibroblast growth factor (bFGF) was obtained from MACGENE (Beijing, China). All reagents were analytical grade, HPLC grade, or the highest pharmaceutical grade available.

### Cell culture

Z310 choroidal epithelial cells were cultured as previously described (Zheng *et al*. 2014; Zheng and Zhao 2002). Briefly, the cells were grown in DMEM supplemented with 10% FBS, 100 units/mL penicillin, 100 μg/mL streptomycin, and 10 μg/mL of gentamycin in a humidified incubator with 95% air and 5% CO_2_ at 37 °C. Trypsin-EDTA was used to digest cells during subculture and Z310 cells were passaged (1:12–16) twice a week. When treated with Pb, 0, 2.5, 5, 10, 15, 20, 50 or 100 μmol/L Pb(AC)_2_ was added to the cells 24 h after initial seeding, and the following studies were performed. The rat brain microvascular endothelial cell line RBE4 was cultured as previously described (Song *et al*. 2014).

### Electron microscopic AMG

Z310 cells (in 100-mm plates) were seeded with a density of 1 × 10^6^ per plate and treated with 10 μmol/L Pb for 24 h. Cells were collected into 1.5-mL EP tubules after digestion with trypsin-EDTA and incubated with 5% bovine serum albumin (BSA) on ice for 10 min. Supernatants were discarded after centrifugation (350 *g* for 10 min). Cell pellets were fixed with 3% glutaraldehyde (GA) for 3 h at room temperature. After treatment of 1% sodium sulfide solutions (11.79 g sodium sulfide nonahydrate and 13.7 g sodium dihydrogen phosphate dihydrate dissolved up to 1000 mL distilled water) for 7 min, AMG developer was used to incubate the pellets at 26 °C for 30 min. The AMG developer consists of 60 mL 50% gum arabic solution, 10 mL sodium citrate buffer (25.5 g citric acid hydrate and 23.5 g sodium citrate dihydrate dissolved up to 100 mL distilled water), 15 mL reductor (0.85 g hydroquinone in 15 mL distilled water at 40 °C) and 15 mL silver nitrate solution (0.12 g silver lactate in 15 mL distilled water at 40 °C). The developer was prepared by mixing the four parts immediately before use (Danscher 1981; Danscher and Stoltenberg 2006). The gum arabic, a protecting colloid in the silver lactate developer, is essential when developing time exceeds 30 min.

The process was stopped by 5% sodium thiosulphate solutions for 10 min. The samples were then post-fixed with 0.5% osmium tetroxide in 0.1 mol/L phosphate buffer (PB) for 1 h, dehydrated in graded series of ethanol, then in propylene oxide, and finally flat-embedded in Epon 812 (SPI-CHEM, West Chester, PA, USA). Ultrathin sections (50–70 nm) were cut with an ultramicrotome (EM UC6, Leica) and mounted on copper grids (6–8 sections/grid). Sections were then counter-stained with uranyl acetate and Pb citrate, and observed under a JEM-1230 electron microscopy (JEOL LTD, Tokyo, Japan). Electron micrographs were captured by the Gatan digital camera and its application software (832 SC1000, Gatan, Warrendale, PA, USA). Operative skills are further illustrated by a diagrammatic sketch in Fig. 1.

### Cell preparation for transmission electron microscopy (TEM)

Z310 cells were washed three times with PBS followed by digestion with 0.25% trypsin-EDTA for 4 min. Cells were collected into microcentrifuge tubes, followed by centrifugation (350 *g* for 10 min). Cell pellets were fixed with 3% GA for 3 h at room temperature. The samples were fixed with 0.5% osmium tetroxide in 0.1 mol/L PB for 1 h, dehydrated in graded series of ethanol, then in propylene oxide, and finally flat-embedded in Epon 812. Ultrathin sections (100 nm) were cut with the ultramicrotome and mounted on nickel mesh grids (6–8 sections/grid). Hitachi electron microscopy (H-7650; Hitachi Ltd, Tokyo, Japan) was used for observation of sections and EDS that can be used to calculate the constituent ratio including weight percent (Wt%) and atom percent (At%) of metallic elements.

### Subcellular fractionation

According to the manufacturer’s directions, cells were fractionated into cytosol, membrane/organelle, nucleus and cytoskeleton using a ProteoExtract^®^ Subcellular Proteome Extraction Kit (Merck Millipore, German). The constituent ratios of protein in such four fractionations were calculated through the product of protein concentrations and corresponding volumes. Protein concentrations were analyzed with Pierce^®^ BCA Protein Assay Kit (Thermo Scientific, USA).

### Cell Pb concentrations measurement

After Pb treatment, cells were collected into 1.5-mL EP tubules. Concentrated nitric acids were utilized for nitrolysis at 100 °C for 1 h. Pb concentrations were standardized by cell numbers. Before detection, we created a calibration curve and ensured that the concentrations were distributed within the linear range. Aliquots of nitrolysis products were used for measurement of cell Pb concentrations expressed as micrograms per deciliter (μg/L) by graphite furnace atomic absorption spectroscopy (AAS) (PinAAcle-Model 900T-Atomic Absorption Spectrometer, PerkinElmer, USA).

### Isolation of B-lymphocytes

Human peripheral venous blood was donated by our group to isolate B-lymphocytes. The blood was mixed at 3:1 with pre-warmed RPMI 1640 medium in EDTA-containing tubes. The mixture was laid gently onto isometric lymphocyte separation medium. Peripheral blood mononuclear cells including B-lymphocytes were transferred with Pasteur pipettes into 50-mL EP tubes from the ring-shape interphase after centrifugation (slow acceleration, no brake). The cells were washed with RPMI 1640 medium, followed by centrifugation (100 *g* for 10 min; fast acceleration, fast brake).

### Statistical analysis

All data are reported as the mean ± SD from at least three independent biological samples. Statistical significance between multiple groups was performed using one-way analysis of variance (ANOVA) followed by two-tailed paired Student’s *t*-tests to compare individual groups. Values of *p* < 0.01 were considered statistically significant.

### Abbreviations

AAS　　　Atomic absorption spectroscopy

AMG　　 Autometallography

BBB　　　Blood−brain barrier

BCB　　　Blood−CSF barrier

BSDs　　 Black silver deposits

CSF　　　 Cerebrospinal fluid

EDS　　　Energy-dispersive X-ray spectroscope

NMDAR　 N-methyl-D-aspartic acid receptor

TEM　　 Transmission electron microscopy

## Conflict of interest

Han Song, Gang Zheng, Xue-Feng Shen, Zai-Hua Zhao, Yang Liu, Yang Liu, Ying-Ying Liu, Jun-Jun Kang, Jing-Yuan Chen and Wen-Jing Luo declare that they have no conflict of interest.
